# Redox-Sensitivity and Site-Specificity of S- and N- Denitrosation in Proteins

**DOI:** 10.1371/journal.pone.0014400

**Published:** 2010-12-21

**Authors:** Frances L. Jourd'heuil, Anthony M. Lowery, Elaina M. Melton, Sanie Mnaimneh, Nathan S. Bryan, Bernadette O. Fernandez, Joo-Ho Park, Chung-Eun Ha, Nadhipuram V. Bhagavan, Martin Feelisch, David Jourd'heuil

**Affiliations:** 1 Center for Cardiovascular Sciences, Albany Medical College, Albany, New York, United States of America; 2 Department of Molecular and Cellular Physiology, Louisiana State University Health Sciences Center, Shreveport, Louisiana, United States of America; 3 Whitaker Cardiovascular Institute, Boston University School of Medicine, Boston, Massachusetts, United States of America; 4 R&D Planning Department, Cellrion Songdo-dong, Yeonsugu, Incheon, Republic of Korea; 5 Department of Native Hawaiian Health, John A. Burns School of Medicine, University of Hawaii at Manoa, Honolulu, Hawaii, United States of America; 6 Department of Anatomy, Biochemistry, and Physiology, University of Hawaii at Manoa, Honolulu, Hawaii, United States of America; Bauer Research Foundation, United States of America

## Abstract

**Background:**

S-nitrosation – the formation of S-nitrosothiols (RSNOs) at cysteine residues in proteins – is a posttranslational modification involved in signal transduction and nitric oxide (NO) transport. Recent studies would also suggest the formation of N-nitrosamines (RNNOs) in proteins *in vivo*, although their biological significance remains obscure. In this study, we characterized a redox-based mechanism by which N-nitroso-tryptophan residues in proteins may be denitrosated.

**Methodology/Principal Findings:**

The denitrosation of *N*-acetyl-nitroso Trp (NANT) by glutathione (GSH) required molecular oxygen and was inhibited by superoxide dismutase (SOD). Transnitrosation to form S-nitrosoglutathione (GSNO) was observed only in the absence of oxygen or presence of SOD. Protein denitrosation by GSH was studied using a set of mutant recombinant human serum albumin (HSA). Trp-214 and Cys-37 were the only two residues nitrosated by NO under aerobic conditions. Nitroso-Trp-214 in HSA was insensitive to denitrosation by GSH or ascorbate while denitrosation at Cys-37 was evident in the presence of GSH but not ascorbate. GSH-dependent denitrosation of Trp-214 was restored in a peptide fragment of helix II containing Trp-214. Finally, incubation of cell lysates with NANT revealed a pattern of protein nitrosation distinct from that observed with GSNO.

**Conclusions:**

We propose that the denitrosation of nitrosated Trp by GSH occurs through homolytic cleavage of nitroso Trp to NO and a Trp aminyl radical, driven by the formation of superoxide derived from the oxidation of GSH to GSSG. Overall, the accessibility of Trp residues to redox-active biomolecules determines the stability of protein-associated nitroso species such that in the case of HSA, N-nitroso-Trp-214 is insensitive to denitrosation by low-molecular-weight antioxidants. Moreover, RNNOs can generate free NO and transfer their NO moiety in an oxygen-dependent fashion, albeit site-specificities appear to differ markedly from that of RSNOs.

## Introduction

The chemistry associated with the production of nitric oxide (NO) in biological systems provides the foundation from which the diverse functions of NO may be interpreted. The nitrosation – i.e. the addition of a nitrosonium (NO^+^) equivalent – of the sulfhydryl group of cysteine residues in proteins to form S-nitrosothiols (RSNOs) has received increasing attention [1] with evidence of its occurrence *in vivo* and demonstration of regulatory effects in various proteins including GTPases [2], proteases [3], ion channels [4], phosphatases [5], and transcription factors [6]. In contrast, the N-nitrosation of primary and secondary amines to form N-nitrosamines (RNNOs) has been studied mainly in the context of carcinogenesis with little attention given to protein N-nitrosation [7]. In 1996, Loscalzo et al. established that tryptophan in albumin is nitrosated by acidified nitrite to form N-nitrosotryptophan [8] but the significance of these findings for the formation of N-nitrosated proteins *in vivo* has remained unclear. Other studies indicate that RNS production *in vivo* gives rise to the formation of NO adducts with the chemical signature of RNNOs [9]–[12]. Collectively, this suggests that – in addition to cysteine - amino acids such as Trp might be nitrosated *in vivo* to form protein RNNOs.

The denitrosation or removal of the NO moiety from amino acid residues is essential for protein RSNOs and RNNOs to function as NO storage or signaling intermediates. Although denitrosation pathways have been detailed for RSNOs [13], information about similar reactions for RNNOs is scarce. Understanding the denitrosation of protein RNNOs in complex biological matrices would help develop and improve methods to detect and identify these molecules, and unravel their biological function. N-nitrosamines in general undergo denitrosation through thermal and photochemical homolytic cleavage of the N-NO bond [14], [15]. In the presence of a nucleophile, denitrosation occurs through the direct or indirect - proton catalyzed - transfer of the nitrosonium group to the nucleophile itself [16]. Kirsch and Korth have proposed that the denitrosation of N-nitrosotryptophan and other derivatives also occurs without proceeding through the proton-catalyzed reaction [17]. In this case, the nitrosamine directly acts as an electrophilic nitrosating agent such that denitrosation of N-nitrosotryptophan by low molecular weight thiols including GSH is dominated by the transnitrosation of GSH and the accumulation of GSNO ([18]). Alternatively, de Biase et al. have proposed that denitrosation in the presence of ascorbate occurs through initial homolytic cleavage of the N-NO bond in nitrosamines [19]. In the present study, we show that the denitrosation of nitrosated Trp residues by excess GSH is driven by the formation of superoxide derived from the oxidation of GSH to GSSG. Transnitrosation occurs only in the absence of oxygen or upon scavenging of superoxide. We also studied the S- and N-denitrosation of human serum albumin and found no evidence for GSH or ascorbate-dependent denitrosation of the N-nitrosated Trp residue indicating that the site of N-nitrosation (Trp-214) is not accessible to low-molecular-weight antioxidants.

## Results

### Denitrosation of N-acetyl nitroso Trp by GSH forms S-nitrosoglutathione only in the absence of molecular oxygen or presence of superoxide dismutase

**Figure 1:**
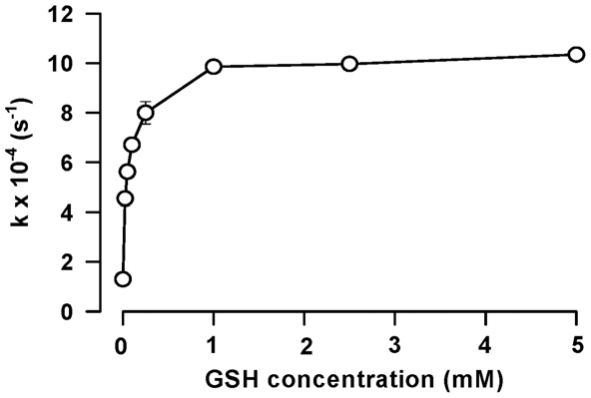
Denitrosation of N-acetyl-nitroso-Trp (NANT) by glutathione (GSH). The decomposition of NANT (100 µM) was followed spectrophotometrically at 335 nm upon incubation with increasing concentrations of GSH in 100 mM phosphate buffer (pH 7.4) containing 100 µM DTPA. NANT decay followed apparent first order kinetics and k_obs_ for NANT decomposition was plotted as a function of [GSH]. The values represent the mean ± SEM (n = 4).

The denitrosation of nitrosated Trp residues can be modeled by studying the stability of N-acetyl nitroso Trp (NANT) in solution. In this set of experiments, NANT decomposition was followed spectrophotometrically at 335 nm upon incubation of 100 µM NANT with various concentrations of GSH in 100 mM phosphate buffer (pH 7.4) containing 100 µM DTPA. As previously shown [18], NANT decay was increased upon addition of GSH and followed apparent first order kinetics. The rate of NANT decay increased with GSH until zero-order dependence was established at 1 mM GSH and above (Figure 1). The apparent rate of NANT decomposition with 2.5 mM GSH was 9.97±0.08×10^−4^. s^−1^ (n = 4), in accordance with values produced for other nucleophiles [16].

**Figure 2:**
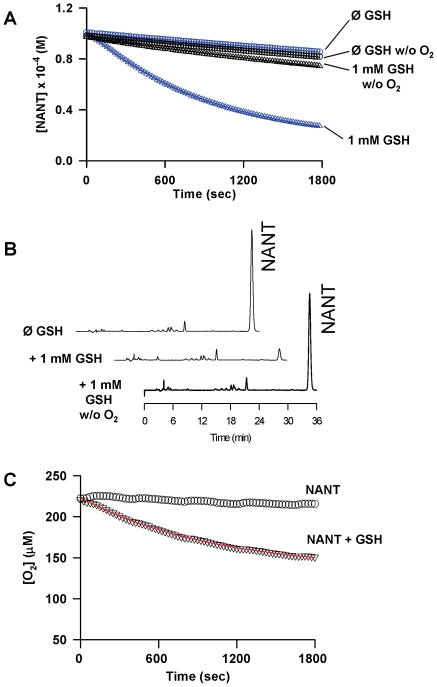
The denitrosation of N-acetyl-nitroso-Trp (NANT) by glutathione (GSH) requires molecular oxygen. (**A**), Time-course of GSH-induced NANT decomposition in the presence or absence of molecular oxygen; representative of at least 4 experiments. (**B**), Representative chromatogram (detection at 335 nm) obtained from the reaction of 100 µM NANT with 1 mM GSH and O_2_. Chromatograms are representative of three independent experiments. (**C**), Time-course of O_2_ consumption in the presence of 100 µM NANT and 1 mM GSH; representative of 4 experiments. The solid lines represent the nonlinear regression fitting of data points (open symbols).

In contrast with a previous study [18], we found that the direct transnitrosation between NANT and GSH could be ruled out as the primary mechanism for NANT decomposition because the disappearance of NANT was inhibited by the exclusion of molecular oxygen (Figure 2A). This was confirmed by reversed phase HPLC, by showing inhibition of GSH-sensitive decomposition of NANT upon deoxygenation (Figure 2B). The HPLC results also revealed close to 90% decomposition of NANT with 1 mM GSH. A portion of the residual absorbance at 30 min observed in the spectrophotometric assay was due to products absorbing at 335 nm derived from the preparation of NANT from acidic nitrite and N-acetyl Trp. Based on the spectrophotometric and HPLC results and an ε of 6100 M^−1^.cm^−1^ at 335 nm [20], NANT concentration decreased by 73.7±1.5 µM (n = 16) over the 30 min incubation period in the presence of 1 mM GSH. Under the same conditions, O_2_ consumption followed apparent first order kinetic such that 67.0±5.3 µM O_2_ was consumed within 30 min (n = 4; Figure 2C).

**Figure 3:**
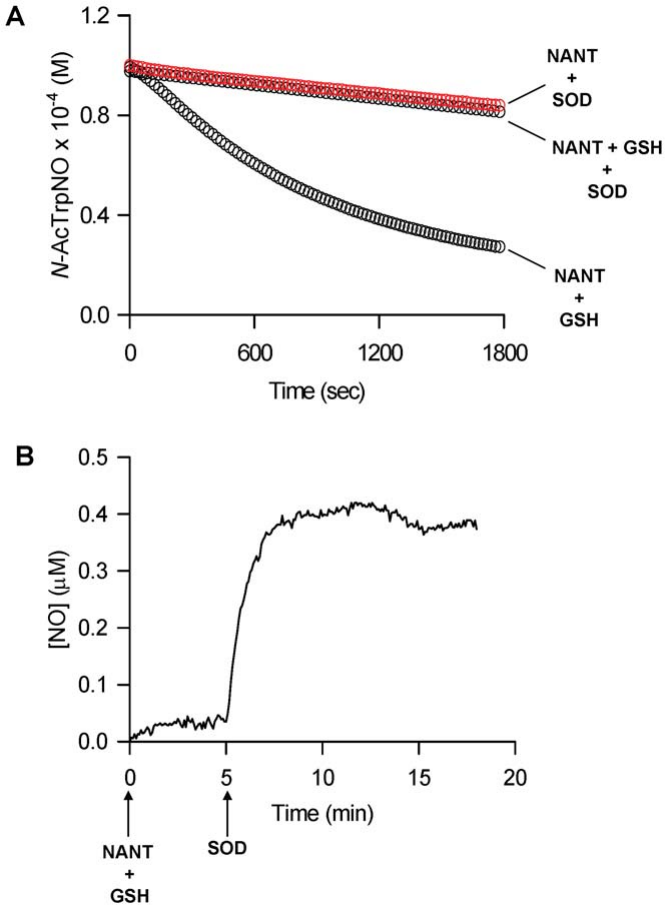
Effect of superoxide dismutase (SOD) on the GSH-induced decomposition of NANT and NO production. (**A**), Time-course of NANT decomposition in the presence of GSH with or without SOD; representative of three experiments. (**B**), NO formation in solution upon incubation of NANT with GSH and SOD; representative of 4 experiments.

Next, we found that superoxide dismutase (SOD) inhibited NANT decomposition by GSH in a fashion similar to that observed in the absence of O_2_ (Figure 3A). Reductive cleavage of NANT by SOD as previously shown for S-nitrosothiols [21], [22] did not occur because addition of SOD alone to NANT did not have any effect (Figure 3A). Low levels of NO were generated in the presence of 100 µM NANT and 1 mM GSH. This was dramatically increased upon addition of SOD, with steady-state levels of NO generation approaching 400 nM (Figure 3B).

**Figure 4:**
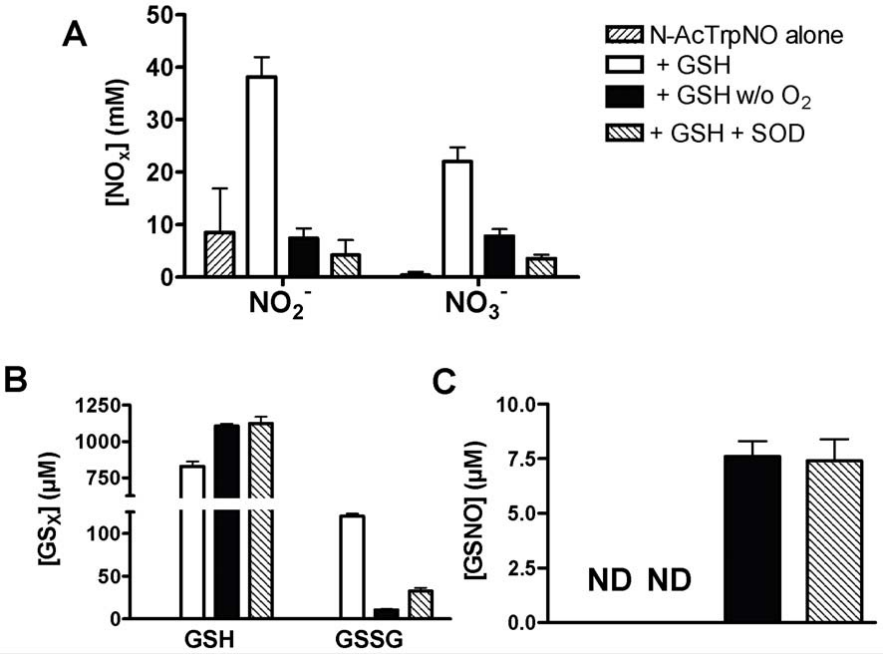
Formation of nitrite (NO_2_^−^) and nitrate (NO_3_^−^, panel A), glutathione disulfide (GSSG, panel B), and S-nitrosoglutathione (GSNO, panel C) during NANT decomposition. *N*-acetyl nitroso Trp (100 µM) was incubated with GSH 1 mM for 30 min in the presence or absence of O_2_ and SOD at 37°C and product formation was determined by anion pairing HPLC. The values represent the mean ± SEM (n = 5–9); ND  =  not detectable.

Finally, GSH, glutathione disulfide (GSSG), GSNO, nitrite (NO_2_^−^), and nitrate (NO_3_^−^) formation were analyzed by ion-pairing HPLC (Figure 4). We found that only NO_2_^−^ was formed with NANT alone while both NO_2_^−^ and NO_3_^−^ were formed upon coincubation of NANT with GSH. In the latter case, there was good agreement between NANT and O_2_ consumption, and NO_2_^−^ + NO_3_^−^ produced amounted to a total of 60.2±5.9 µM (n = 9). Glutathione disulfide was formed in excess of NANT consumed or NO_2_^−^ + NO_3_^−^ produced, totaling 120.2±2.8 µM (n = 9). There was no evidence for GSNO formation in the presence of GSH unless SOD was present or O_2_ absent. In either case, the yields of GSNO amounted to approximately 30% of the total amount of NANT decomposed.

### Site-specific nitrosation and denitrosation of human serum albumin

**Figure 5:**
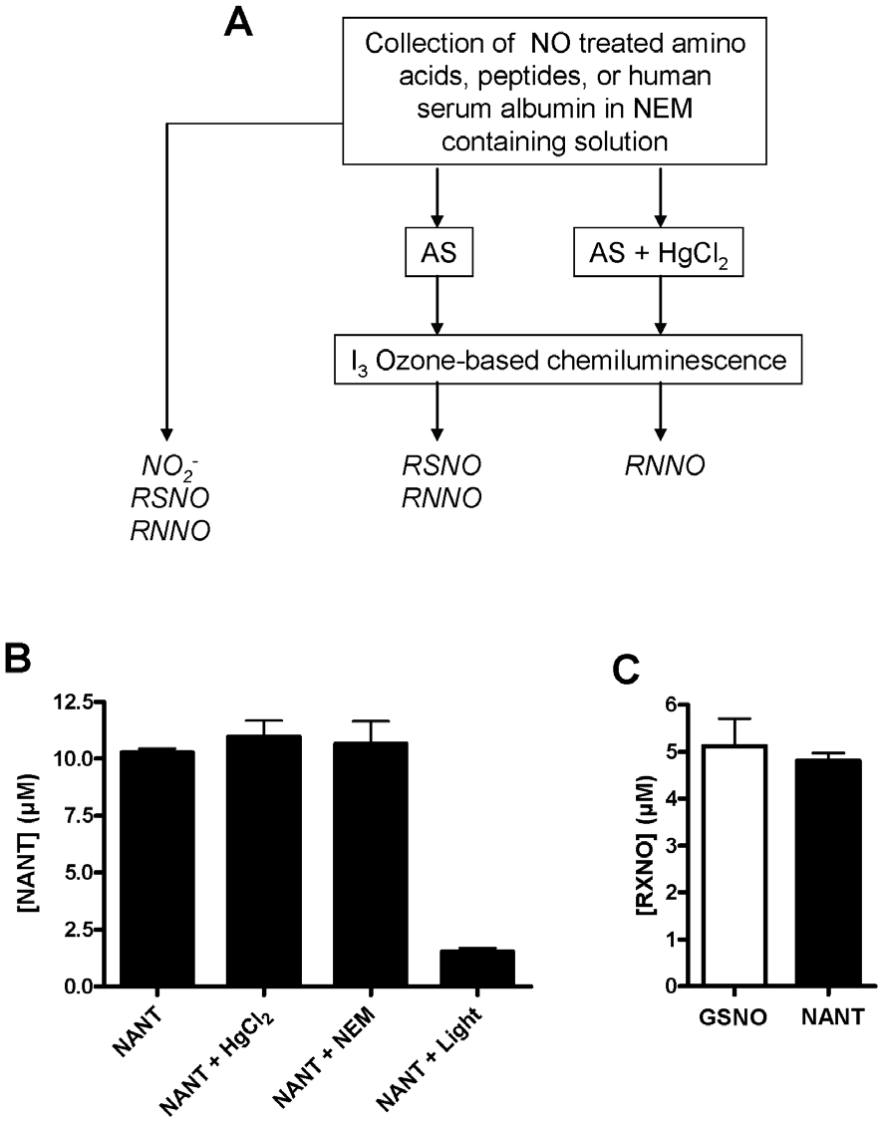
Validation of nitrosamine determination by tri-iodide-based chemiluminescence assay. (**A**), Flow chart for the determination of nitroso species using the tri-iodide chemiluminescence assay. Details may be found under Materials and Methods. (**B**), N-acetyl-nitroso-Trp (NANT, 10 µM) was preincubated with HgCl_2_ or NEM in the presence of acidified sulfanilamide. The samples were then quantified by gas phase chemiluminescence as described under Materials and Methods. The values represent the mean ± SEM (n = 3). (C), Stock solutions of GSNO and NANT of known concentrations were diluted and mixed together in 100 mM phosphate buffer containing DTPA (100 µM) and nitrite (40 µM) to obtain a final concentration of 5 µM for each compound. Concentrations were immediately determined using the same tri-iodide based chemiluminescence assay (n = 3, mean ± SEM).

To evaluate the impact of GSH-induced denitrosation of nitrosated Trp residues in human serum albumin (HSA), we established first that tryptophan and cysteine were the only proteinogenic residues to form nitrosated products in significant amounts upon incubation with NO in oxygenated solution (Table 1). Next, RSNO and RNNO content in nitrosated HSA was determined. This was accomplished using a chemiluminescence-based assay as previously described [23] (Figure 5A). In this assay, acidic tri-iodide (I_3_^−^) reduces RSNOs, RNNOs, and NO_2_^−^. The samples are pretreated with acidified sulfanilamide to eliminate NO_2_^−^ by conversion to a diazonium cation. The RSNO concentration is determined by measuring the difference in chemiluminescence signal between samples pretreated with and without HgCl_2_. Mercuric ions cleave the S-NO bond in RSNOs without affecting the signal obtained from RNNOs such that in a RSNO/RNNO mixture the remaining signal after pretreatment with HgCl_2_ is derived exclusively from the RNNO component (Figure 5A). To confirm the validity of the approach within the specific context of the present experiments, NANT (10 µM) was preincubated with HgCl_2_ or NEM, all in the presence of acidified sulfanilamide. The samples were then injected into a purge vessel containing the tri-iodide mixture and the amount of NO evolving from the purge vessel was quantified by gas phase chemiluminescence as described under Materials and Methods. As illustrated in Figure 5B, it was evident that NANT is decomposed only upon light irradiation. In a different set of experiments, stock solutions of GSNO and NANT of known concentrations were diluted and mixed together in 100 mM phosphate buffer containing DTPA (100 µM) and nitrite (40 µM) to obtain a final concentration of 5 µM for each compound. Concentrations were immediately determined using the same tri-iodide based chemiluminescence assay. There was no statistical difference between the different values (Figure 5C) with recovery corresponding to 102 (SEM = 11, n = 3) and 96 (SEM = 5, n = 3) % for GSNO and NANT, respectively. These results indicate that nitrosated Cys and nitrosated Trp can be detected with comparable efficiency in agreement with previous studies [23]. While concerns have been raised about the specificity of the triiodide assay in complex biological systems, our results indicate that these do not apply to the much simpler in vitro conditions of the present experiments.

**Table 1:** Detector response obtained from the reaction of N-acetylated L-amino acids by NO in oxygenated solution.

Amino Acids	Peak Area (mV.sec)
None (buffer control)	66.6±12.5
Alanine	127.5±73.6
Arginine	41.9±10.4
Asparagine	38.8±5.5
Aspartate	36.8±1.1
**Cysteine**	**11600.0±410.0**
Glutamate	62.6±33.5
Glutamine	32.5±8.7
Glycine	35.4±10.3
Histidine	108.9±53.6
Isoleucine	74.3±31.3
Leucine	35.4±4.0
Lysine	37.7±11.0
Methionine	97.5±26.1
Phenylalanine	87.1±29.9
Proline	36.0±4.5
Serine	34.4±6.6
Threonine	66.3±39.5
**Tryptophan**	**6800.0±50.6**
Tyrosine	55.5±2.8
Valine	189.0±135.2

Acetylated amino acids (1 mM) were incubated for 30 min at 37°C with 20 µM DEA/NO at ambient oxygen concentration in 100 mM phosphate buffer (pH 7.4) containing 100 µM DTPA. Product formation was determined using a chemiluminescence-based assay as described under Materials and Methods. The values represent the mean ± SEM, n = 3.

**Figure 6:**
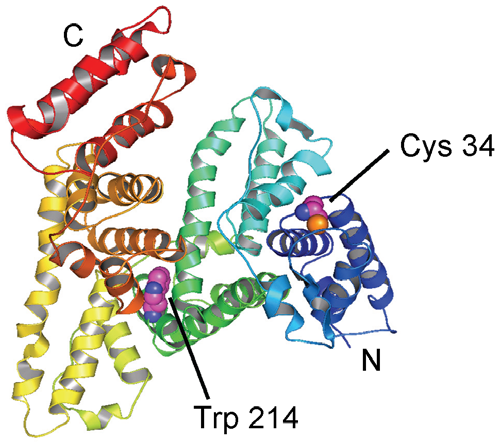
Overview of human serum albumin (HSA). The protein secondary structure is shown schematically and the domains are colored-coded as follows: I, blue; II, green; III, yellow; IV, red. Cys-34 and Trp-214 are shown in a space-filling representation and colored by atom types (PDB ID: 1E78; [45]).

**Figure 7:**
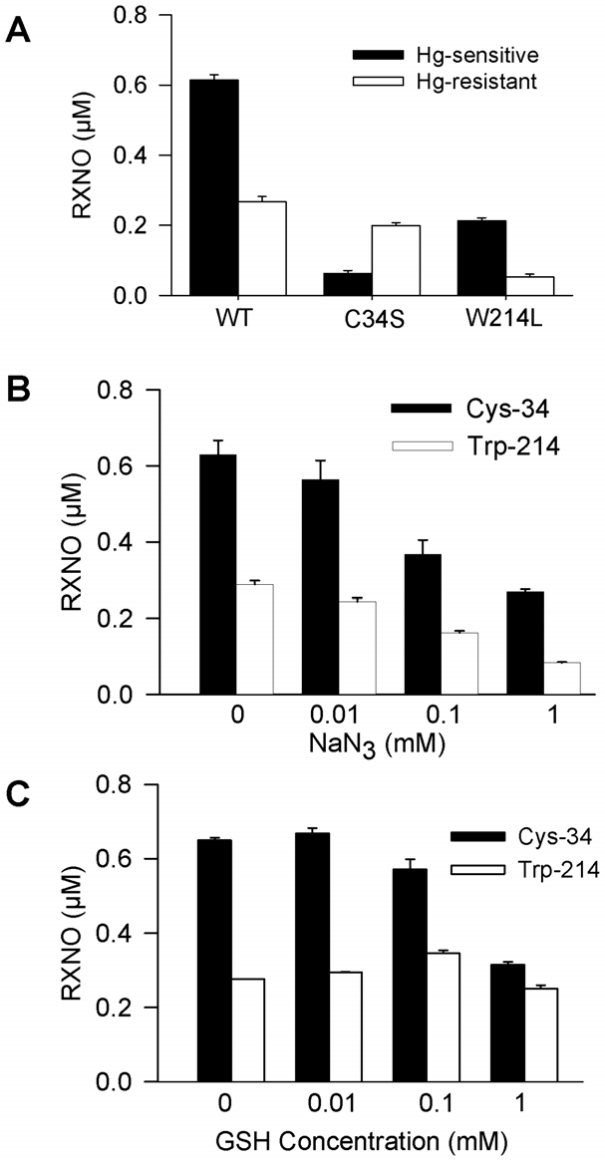
Site-directed nitrosation of human albumin. (**A**), The presence of mercury-sensitive and -resistant species in wild-type (WT), C34S and W214L recombinant HSA (3 mg/ml) treated with 20 µM DEA/NO for 30 min at 37°C was determined by reductive chemiluminescence. The effect of sodium azide (**B**) and GSH (**C**) on the amount of nitrosated Cys-34 and Trp-214 was determined after desalting of the samples. The values represent the mean ± SEM (n = 3).

Human serum albumin contains only one tryptophan residue (Trp-214) and one reduced cysteine residue (Cys-34; Figure 6). Evidence that Trp-214 and Cys-34 were the sites of nitrosation by NO/O_2_ was attained by generating recombinant proteins in which serine replaced Cys-34 (C34S) and lysine replaced Trp-214 (W214L). These recombinant and the wild type (WT) proteins were treated for 30 min at 37°C with 20 µM DEA/NO in 100 mM phosphate buffer (pH 7.4) containing 100 µM DTPA. The results illustrated in Figure 7A indicated that removal of Trp-214 in HSA essentially eliminated the mercury-resistant signal, while removal of Cys-34 eliminated the mercury sensitive-signal. Overall, these results indicated that the primary sites of nitrosation in HSA are Trp-214 and Cys-34. Although the concentration of mercury-resistant signal obtained from WT HSA was of the same order as that obtained with C34S, the concentration of mercury-sensitive signal in W214L amounted to only a third of the signal recovered from WT (Figure 7A). The reasons for these results remain unclear in as much as opposite observations were made when acidified nitrite was used, i.e. an increase in the mercury-sensitive signal with W214L relative to WT [24].

Azide inhibited HSA nitrosation in a concentration-dependent manner such that, in the presence of 1 mM azide, Cys-34 and Trp-214 nitrosation was inhibited by approximately 57% and 71%, respectively (Figure 7B). In addition, we noted a marked difference in sensitivity of the two residues to the presence of GSH (Figure 7C). Upon incubation with 1 mM GSH, nitrosation yields of Cys-34 were inhibited by approximately 50%, but the yields of nitroso-Trp-214 remained unchanged. In contrast, GSH (1 mM) inhibited by approximately 80% the nitrosation of *N*-acetyl-Trp (200 µM) by 20 µM DEA/NO (1.36±0.11 µM NANT formed in the absence of GSH vs. 0.25±0.03 µM in the presence of GSH; *P*<0.01, n = 3).

**Figure 8:**
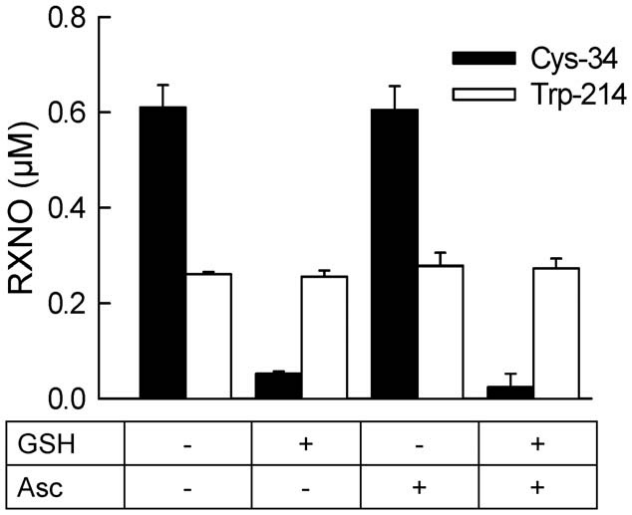
Stability of nitrosated human serum albumin in the presence of GSH and ascorbate. HSA (3 mg/ml) was incubated with 20 µM DEA/NO in 100 mM phosphate buffer (pH 7.4) containing 100 µM DTPA for 30 min at 37°C. Samples were then incubated for an additional 30 min either alone or in the presence of GSH (1 mM) and ascorbate (Asc; 300 µM) in 100 mM phosphate buffer (pH 7.4) containing 100 µM DTPA, after which the samples were desalted. Nitrosation at Cys-34 and Trp-214 was then determined using reductive chemiluminescence. The values represent the mean ± SEM (n = 3).

**Figure 9:**
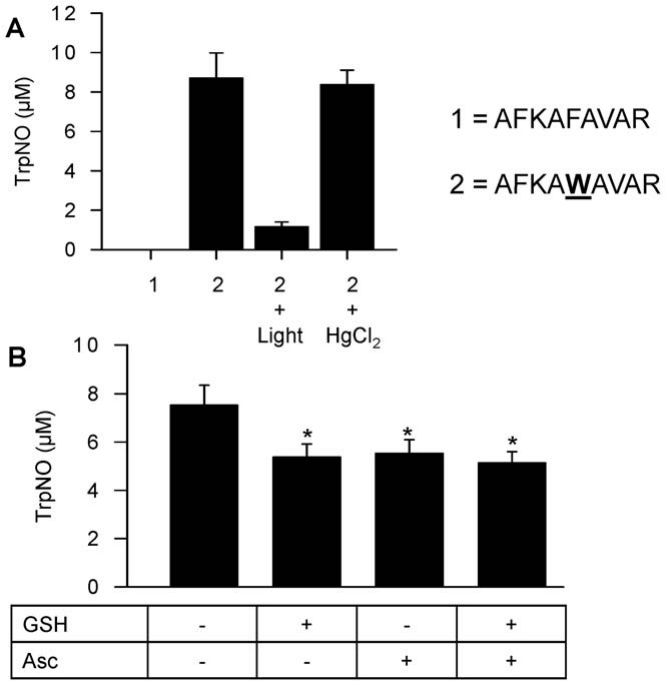
Stability of nitrosated fragment of helix IIa-h2 of HSA containing Trp-214. **A**, A peptide (#2) corresponding to the fragment of helix IIa-h2 of HSA was nitrosated using acidified nitrite. The peptide was then diluted to obtain a final concentration of nitroso Trp of 10 µM (based on an ε of 6100 M^−1^.cm^−1^ at 335 nm) and the stability of the nitrosated peptide was determined by reductive chemiluminescence. There was no evidence for an NO-dependent signal in the control peptide (#1) that did not contain the Trp residue but that was exposed to acidified nitrite. Peptide #2 (10 µM) was also incubated in the presence of light for 30 min or with HgCl_2_ (final concentration 4.9 mM) for 15 min. The values represent the mean ± SEM (n = 3). **B**, The nitrosated peptide (10 µM) was incubated for 30 min either alone or in the presence of GSH (1 mM) and ascorbate (Asc; 300 µM) in 100 mM phosphate buffer (pH 7.4) containing 100 µM DTPA. Nitrosation of Trp was then determined using reductive chemiluminescence upon pretreatment of paired samples with or without HgCl_2_ to elimate the contribution of RSNOs and as described under Materials and Methods. The values represent the mean ± SEM (n = 3); *p<0.05 vs. peptide alone.

To evaluate possible denitrosation of HSA, the protein was incubated with DEA/NO for 30 min, incubated with either GSH or ascorbate for an additional 30 min, and then desalted before nitroso species determination. Incubation of nitrosated WT HSA with GSH but not ascorbate eliminated the signal associated with Cys-34 (Figure 8). There was no effect of these treatments on nitroso-Trp-214, in contrast to the results obtained with NANT (Figure 2). To obtain further evidence that the localization of Trp-214 within the hydrophobic core of HSA limited the denitrosation of nitroso-Trp by GSH, we evaluated the stability of nitroso-Trp in a peptide consisting of the portion of helix II that contains Trp-214. We found no evidence for nitrosated products in a peptide (#1, Figure 9A) that did not contain the Trp residue and submitted to nitrosation with acidified nitrite. In contrast, light irradiation decreased by more than 80% the signal obtained from the Trp-214 containing peptide (#2) while mercury did not have any effect on the signal, consistent with nitroso-Trp formation. In agreement with our hypothesis, we found that incubation for 30 min of the nitrosated peptide with either GSH or ascorbate resulted in a significant decrease in the nitroso-Trp signal (Figure 9B).

### Transnitrosation between NANT and cellular proteins

**Figure 10:**
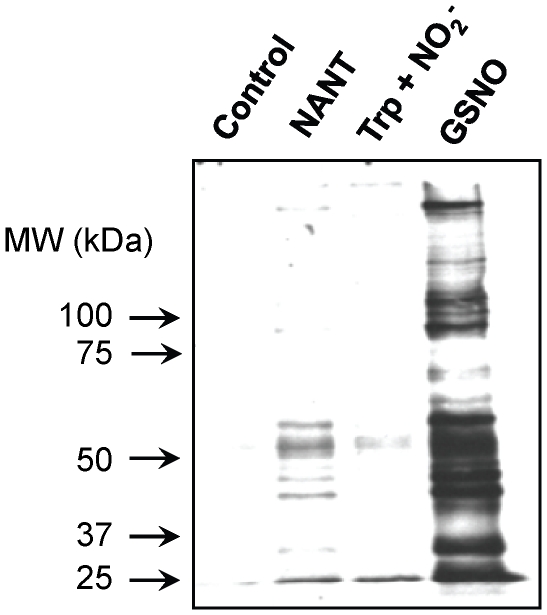
Transfer of NO between nitroso-*N*-acetyl-Trp and proteins. Cell lysates obtained from murine fibroblasts were incubated alone (Control), with 10 µM nitroso-*N*-acetyl-Trp (NOTrp), 10 µM sodium nitrite and *N*-acetyl-Trp (Trp + NO_2_^−^), or 10 µM GSNO (GSNO) for 1 hr at 37°C. Nitrosated proteins were then determined using the biotin switch assay as described under Materials and Methods. Equal loading was verified using Coomassie blue staining. The immunoblot is representative of three experiments.

S-nitrosocysteine and other low molecular weight RSNOs have been used as alternates to NO donors in order to nitrosate cellular proteins [25]. The observation of slow but efficient transnitrosation between NANT and GSH in the presence of SOD led us to entertain the possibility that NANT may also be used to transnitrosate proteins in the cellular environment where SODs are abundant. To this end, 10 µM NANT was incubated with cell lysates obtained from mouse fibroblasts for 1 hr at 37°C and S-nitrosated proteins were examined using a biotin switch assay as described under Materials and Methods. Coincubation of the cell lysates with NANT resulted in a banding pattern of nitrosated proteins that was distinct from the one obtained upon treatment with GSNO (Figure 10). The extent of protein nitrosation was less compared to GSNO, and addition of *N*-acetyl-Trp with nitrite (NO_2_^−^) resulted in only minimal immunoreactivity.

## Discussion

**Figure 11:**
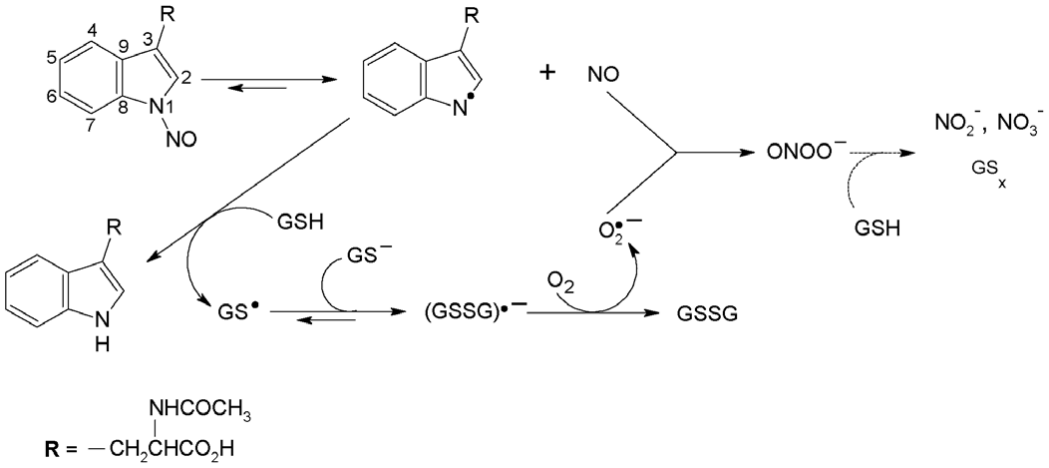
Proposed pathway for the denitrosation of NANT by GSH in the presence of molecular oxygen.

The denitrosation of NANT occurs through nucleophilic attack on the protonated form of the nitrosamine, and the reversibility of the reaction is suppressed upon addition of a trap for the NO species liberated [16]. More recent work had indicated that NANT denitrosation by GSH occurred primarily through transnitrosation to form Trp and GSNO [18]. In contrast, we here show that transnitrosation cannot account for GSH-sensitive NANT denitrosation because it is almost completely inhibited upon deoxygenation. The absence of GSNO formation could not be explained through secondary reactions of GSNO with excess GSH or superoxide because the decomposition of micromolar concentrations of GSNO even in the presence of 1 mM GSH or excess superoxide is far too slow [26], [27]. In contrast, GSNO formation was evident in the absence of oxygen. Additional product characterization revealed stoichiometric amounts of NANT and O_2_ consumed. The denitrosation of NANT by GSH and the formation of GSSG were almost completely inhibited by SOD while an increase in free NO was detected upon addition of SOD. A potential explanation is that superoxide might be formed from contaminating trace metals. However, this is unlikely because all of our experiments were done in the presence of the metal chelator DTPA. The simplest explanation consistent with all these results is a reaction pathway that involves the homolytic cleavage of NANT to form a Trp indole radical and free NO (Figure 11). This is followed by the reduction of the Trp indole radical by GSH and reaction of the thiyl radical formed with excess GSH to yield a glutathione disulfide anion radical. In the presence of excess GSH, the reaction is driven by the rapid removal of the disulfide radical anion through reduction of O_2_ to superoxide [28] that combines with free NO to form peroxynitrite. The initial step in this pathway would be similar to the mechanism of denitrosation of *N*-nitrosomelatonin by ascorbate proposed by De Biase et al. [19]. The fact that superoxide dismutase inhibits the increase in the rate of denitrosation by GSH indicates that superoxide itself plays an important role in driving the homolytic cleavage of the N-NO bond, possibly by limiting the recombination of NO with the aminyl radical. In the absence of O_2_ but with excess GSH, GSNO may be formed through radical-radical combination of NO with the thiyl radical [29], upon proton-catalyzed denitrosation of NANT, or through direct transnitrosation as previously described [16], [18]. In our hands, NANT denitrosation in the absence of GSH was insensitive to O_2_ suggesting proton-catalyzed denitrosation in the presence of excess phosphate or direct transnitrosation. As clearly illustrated earlier by Kytzia et al. in the case of ascorbate, the outcome of N-acetyl nitroso Trp derivatives denitrosation is dependent on secondary reactions that impact the formation of free NO [30].

Human serum albumin is unique among other mammalian orthologs as it contains only one reduced cysteine (Cys-34) and one tryptophan residue (Trp-214) [31]. Cysteine-34 is located at the surface of the protein in a loop between helices Ia-h2 and Ia-h3 (Figure 6), and is conjugated to cysteine or glutathione in 30–40% of HSA molecules in the circulation. The 36 other cysteine residues present in HSA are oxidized to form 18 disulfide bridges that are not accessible for nitrosative modification. The indole group of Trp-214 projects from helix IIa-h2 that forms one side of the drug 1 binding site. Using recombinant proteins lacking either Cys-34 or Trp-214, we showed that these two residues were the principal targets of nitrosation in the NO/O_2_ system in accordance with a previous report demonstrating similar results with acidified nitrite [24]. The nitrosation of Trp-214 by NO/O_2_ was sensitive to azide, consistent with an N_2_O_3_-mediated process, although part of the decrease could be due to azide-mediated denitrosation [16]. Glutathione also inhibits *N*-acetyl-Trp nitrosation [20] but there was no effect on the nitrosation of Trp-214 (Figure 7). Nedospasov and coworkers have proposed that the hydrophobic interior of albumin serves as a catalyst for NO autoxidation such that the rate of Trp nitrosation for albumin is much faster compared to free Trp [32]. The size and hydrophilic nature of GSH would forbid direct contact of GSH with the hydrophobic environment surrounding Trp-214. This would provide conditions for which the efficacy of GSH as a competitive scavenger of Trp-214 nitrosation relative to *N*-acetyl-Trp would be diminished, and explain the absence of effect of GSH on Trp-214, at least in the presence of a 5-fold excess of GSH (200 µM albumin vs. 1 mM).

The location of Cys-34 and Trp-214 in distinct regions of HSA also offered a testable paradigm related to the stability of the nitroso species associated with HSA. In short, although nitrosated Trp residues are susceptible to denitrosation by GSH, the solubility of GSH (and other low molecular weight thiols) would preclude access to Trp-214 buried within one of the hydrophobic pockets of HSA. Conversely, Cys-34 is accessible to GSH (as evidenced by its thiolation *in vivo*) and the stability of nitrosated Cys-34 should be greatly affected by GSH. The lack of effect of ascorbate in contrast to NANT [30] and a peptide containing Trp-214 (Figure 9) illustrates the poor sensitivity of S-nitrosated HSA to this antioxidant unless it is used at supraphysiological concentrations or in the presence of certain metals [33]. These findings offer the intriguing possibility that the relative amount of RSNOs and RNNOs associated with HSA is regulated by circulating biomolecules that have differential access to Cys-34 and Trp-214. In this context, recent studies have revealed that fatty acid binding to HSA increases its S-nitrosation and associated antibacterial and cytoprotective properties [34], [35]. Possible NO transfer between Trp-214 and associated fatty acids may need to be considered in view of studies indicating that nitrated fatty acids are found in relative abundance in plasma [36]. It is also possible that a fraction of S-nitrosated Cys on albumin is formed by transnitrosation from N-nitrosated Trp although the two nitrosation sites – Cys-34 and Trp-214 – are far apart.

In summary, several conclusions may be drawn from our results:

1. The denitrosation of N-nitrosated Trp derivatives in the presence of excess GSH occurs through homolytic cleavage of the N-NO bond driven by the oxidation of GSH and formation of superoxide and peroxynitrite (Figure 11).
2. As illustrated with HSA, the primary factor limiting RNNO denitrosation by low molecular weight antioxidants such as GSH is the steric accessibility of the nitrosated Trp; as a result, only appropriately located residues within proteins should be available for denitrosation. This issue was previously conceptualized with regard to Trp nitrosation and should also be considered as far as protein RNNO denitrosation is concerned [37].
3. Techniques for the determination of RNNOs such as chemiluminescence-based assays require preincubation periods that may lead to thiol induced degradation of RNNOs [23]. Removal of low molecular weight thiols through desalting or alkylation of sulfhydryl groups using NEM or iodoacetamide for example should be strategies to consider in order to minimize artefactual degradation of RNNOs.
4. The denitrosation of nitrosated Trp residues by GSH is slow and should allow for the accumulation of significant amounts of RNNOs *in vivo*. This should be most evident in the extracellular environment, which contains relatively low antioxidant concentrations and may allow accumulation of RNNOs associated with the extracellular matrix in tissues or plasma proteins in the circulation [10]. This important issue still awaits additional experimental confirmation.
5. Efficient transnitrosation from N-nitrosated Trp derivatives to GSH (or other low molecular thiols) does not occur unless molecular oxygen is absent or SOD present. Thus, RSNO formation *in vivo* upon pharmacological delivery of N-nitrosated Trp derivatives should be favored in hypoxic or anoxic tissues. Similarly, our results indicate that N-nitrosated Trp derivatives may potentially be used as alternatives to GSNO/S-nitrosocysteine for the intracellular delivery of transnitrosating agents and S-nitrosation of proteins in the SOD and GSH-rich intracellular environment. It is still to be determined whether N-nitrosated Trp derivatives such as NANT can be transported across the plasma membrane as efficiently as S-nitrosocysteine [25].

## Materials and Methods

### Materials

DEA-NONOate (DEA/NO) was obtained from Cayman Chemicals (Ann Arbor, MI). All N-acetylated L-amino acids were purchased from Sigma except for leucine, histidine, and valine which were purchased from Fisher Scientific (Hampton, NH). The peptides AFKAFAVAR and AFKAWAVAR were purchased from GenScript Corp. (Piscataway, NJ). The anti-biotin antibody was from Bethyl Laboratories (Montgomery, TX) and the anti-rabbit HRP antibody from Amersham Pharmacia (Piscataway, NJ). The HRP detection kit was from Pierce (Rockford, IL). The Lipidex-1000 columns were from Packard Instruments. All other chemicals were purchased from Sigma Chemical Co. (St Louis, MO).

### Synthesis and purification of recombinant human serum albumin

Specific mutations were introduced into the HSA-coding region in a plasmid vector containing the entire HSA coding region as previously described [38]–[41]. Mutations in the DNA sequence of the HSA coding region were verified as previously described. The mutated HSA coding regions which contained the native HSA secretion signal sequence were introduced into the yeast species *Pichia pastoris* by homologous recombination. The secreted HSA was isolated from growth medium as follows. The medium was brought to 50% saturation with ammonium sulfate at room temperature. The temperature was then lowered to 4°C, and the pH was adjusted to 4.4, the isoelectric point of HSA in a solution 50% saturated with ammonium sulfate. The precipitated protein was collected by centrifugation and resuspended in distilled water. Dialysis was carried-out for 72 h against 100 volumes of phosphate-buffered saline (PBS; 137 mmol/L NaCl, 2.7 mmol/L KCl, 4.3 mmol/L Na_2_HPO4, 1.4 mmol/L KH_2_PO_4_, pH 7.4) with one change of buffer. The solution was loaded onto a column of cibacron blue immobilized on Sepharose 6B. After the column was washed with 10 bed volumes of PBS, HSA was eluted with 3M NaCl. The eluent was dialyzed against PBS and passed over a column of Lipidex-1000 to remove hydrophobic ligands possibly bound to the HSA [38], [39], [41]. The resulting protein exhibited a single band after sodium dodecyl sulfate-polyacrylamide gel electrophoresis (SDS-PAGE).

### UV-Visible quantification of NANT

The decomposition of NANT (100 µM) at 37°C was followed by monitoring the absorbance change at 335 nm (ε = 6100 M^−1^.cm^−1^ [20]). Measurements were carried out using a Shimadzu UV-1601 PC spectrometer (Shimadzu Scientific Instruments Inc. Columbia, MD, USA). NANT was prepared using acidified nitrite as previously described [20].

### Reaction of NANT and GSH with nitric oxide

In a typical experiment, a one mL reaction volume containing various concentrations of NANT and GSH was incubated in 100 mM phosphate buffer (pH 7.4). After 30 min incubation at 37°C, the samples were prepared for analysis by high performance liquid chromatography or chemiluminescence detection as described below. All experiments were performed in the presence of the metal chelator DTPA (100 µM) and in the absence of light to limit the artefactual decomposition of NANT and the reaction products.

### High performance liquid chromatography (HPLC) analysis of tryptophan reaction products

NANT was directly quantified by reversed-phase HPLC. Samples were injected onto a 250×4.6 mm 5-µm octadecyl silane C_18_ Prevail column isocratically running at a flow rate of 1 ml/min with distilled water containing trifluoro acetic acid (TFA; 0.1%) and acetonitrile. The acetonitrile concentration was increased from 20% to 27% in a linear gradient from 13 min to 38 min after injection, with the reactions products detected at 335 nm.

The products obtained from the denitrosation of NANT by GSH were also studied by ion-pairing HPLC as previously described [42]. Samples were injected onto a 250×4.6 mm 5-µm octadecyl silane C_18_ ultrasphere column (Beckman Coulter, Inc. Fullerton, CA) isocratically running at a flow rate of 1 ml/min with 10 mM K_2_HPO_4_, 10 mM tetrabutylammonium hydrogen sulfate (TBAHS) in acetonitrile-water (5∶95, v/v, pH 7.0). The reaction products of GSH and nitrite and nitrate were detected at 210 nm and the identity of each peak was confirmed by co-elution with authentic standards.

### Detection of nitric oxide

Nitric oxide formation was measured using a Clark-type NO electrode (Iso-NO with 2 mm shielded sensor; WPI, Sarasota, FL). Changes in current output were recorded and NO release was quantified by comparison with a standard curve constructed by addition of increasing concentrations of NaNO_2_ under reducing conditions (KI/H_2_SO_4_).

### Chemiluminescence detection

In a typical experiment, 800 µl of sample was transferred to a glass tube containing 100 µl of 100 mM NEM. The samples was kept on ice and in the dark for 15 min before addition of 100 µl of 100 mM sulfanilamide in 1 M HCl and incubation for another 15 min on ice to scavenge nitrite. Paired samples were also incubated with or without HgCl_2_ (final concentration 4.9 mM) for an additional 15 min to evaluate for the presence of RSNOs. The concentration of nitroso species in the samples was evaluated by quantification of the amount of NO liberated after injection of the sample into a purge vessel containing 4.5 ml of glacial acetic acid and 500 µl of an aqueous mixture comprised of 450 mM potassium iodide and 100 mM iodine [23]. The vessel was kept at 70°C via a water jacket with the solution constantly purged with nitrogen, and changed every four injections. The amount of NO evolving from the purge vessel was quantified by gas phase chemiluminescence (NOA 280: Sievers Instruments; Boulder, CO or CLD 77am sp, Eco Physics, Ann Arbor, MI). Peak integration was performed and results were converted to NO concentrations using authentic NO as a standard.

### Detection of S-Nitrosated Proteins Using Biotin Derivatization Coupled to Western Blotting (Biotin Switch Assay; [43])

Cell lysates were prepared from NIH 3T3 cells [44] and diluted to a final concentration of 1 mg/ml in either 100 mM phosphate buffer and 0.1 mM DTPA (pH 7.4) or HED buffer containing 250 mM Hepes, 1 mM EDTA, and 0.1 mM DTPA (pH 7.7). After treatment with the NO donor, 75 µl of the lysate was loaded onto a Micro Bio-Spin 6 column (Bio-Rad, Hercules, CA) equilibrated with HED and the column was centrifuged at 1,000× g for 5 min. Seven microliters of 25% SDS and 1.5 µl of 20% MMTS in DMF were added to the 75 µl eluate. The sample was then incubated at 50°C in a water bath for 20 minutes with frequent vortexing. Thereafter, samples were desalted to remove MMTS and 8 µl of 2 mM biotin-HPDP in DMSO with 4 µl of 100 mM ascorbate was added. The sample was incubated for 60 min before desalting 4 times and final resuspension into HED containing 0.5% Triton X-100. After SDS-PAGE biotinylated proteins were detected by immunobloting using an anti-biotin antibody as a primary, an anti-rabbit HRP antibody as a secondary, and a HRP detection kit.

### Statistics

For groups of three or more, the data were analyzed by one-way analysis of variance, and when a significant difference was suggested, the Tukey test was used as a post-hoc test. Comparisons restricted to two groups were analyzed using the Student's t-test. A probability value of less than 0.05 was considered to represent a statistically significant difference.
